# Predictors of Infant Age at Enrollment in Early Infant Diagnosis Services in Kenya

**DOI:** 10.1007/s10461-016-1404-z

**Published:** 2016-04-23

**Authors:** Kathy Goggin, Catherine Wexler, Niaman Nazir, Vincent S. Staggs, Brad Gautney, Vincent Okoth, Samoel A. Khamadi, Andrea Ruff, Michael Sweat, An-Lin Cheng, Sarah Finocchario-Kessler

**Affiliations:** 1 Children’s Mercy Hospitals and Clinics, Health Services and Outcomes Research, 2401 Gillham Road, Kansas City, MO 64108 USA; 2 University of Missouri-Kansas City, School of Medicine, Kansas City, MO USA; 3 University of Missouri-Kansas City, School of Pharmacy, Kansas City, MO USA; 4 University of Kansas Medical Center, Department of Family Medicine, Kansas City, KS USA; 5 University of Kansas Medical Center, Department of Preventive Medicine, Kansas City, KS USA; 6 Global Health Innovations, Kansas City, MO USA; 7 Kenya Medical Research Institute, Nairobi, Kenya; 8 Johns Hopkins Bloomberg School of Public Health, Department of International Health, Baltimore, MD USA; 9 Medical University of South Carolina, Department of Psychiatry and Behavioral Sciences, Charleston, SC USA; 10 University of Missouri-Kansas City, School of Nursing and Health Studies, Kansas City, MO USA

**Keywords:** HIV/AIDS, EID, PMTCT, Vertical prevention, Infants, Global health

## Abstract

Despite the importance of early detection to signal lifesaving treatment initiation for HIV+ infants, early infant diagnosis (EID) services have received considerably less attention than other aspects of prevention of mother to child transmission care. This study draws on baseline data from an on-going cluster randomized study of an intervention to improve EID services at six government hospitals across Kenya. Two logistic regressions examined potential predictors of “on time” (infant ≤6 weeks of age) vs. “late” (≥7 weeks) and “on time” versus “very late” (≥12 weeks) EID engagement among 756 mother-infant pairs. A quarter of the infants failed to get “on time” testing. Predictors of “on time” testing included being informed about EID by providers when pregnant, perceiving less HIV stigma, and mother’s level of education. Predictors of “very late” testing (≥12 weeks of age) included not being informed about EID by providers when pregnant and living farther from services. Findings highlight the importance of ensuring that health care providers actively and repeatedly inform HIV+ mothers of the availability of EID services, reduce stigma by frequently communicating judgment free support, and assisting mothers in early planning for accessing EID services. Extra care should be focused on engaging mothers with less formal education who are at increased risk for seeking “late” EID testing. This study offers clear targets for improving services so that all HIV-exposed infants can be properly engaged in EID services, thus increasing the potential for the best possible outcomes for this vulnerable population.

## Introduction

Early infant diagnosis (EID; infant ≤6 weeks of age) of HIV infection that facilitates prompt treatment of HIV+ infants is a critical component of prevention of mother-to-child transmission (PMTCT) efforts. EID is especially important in Kenya where HIV prevalence among pregnant women is 6.2 % [1] and 15 % of infants born to HIV+ mothers become infected [2]. In 2008 the Children with HIV Early ART (CHER) study provided evidence that early antiretroviral therapy (ART) initiation (before 12 weeks) reduced mortality by 76 % and slowed the progression of HIV by 75 % by improving long-term viral suppression and weight for age scores [3]. These findings prompted new World Health Organization (WHO) guidelines to treat all HIV+ infants under age 2 rather than waiting for immunologic thresholds to be reached [4]. Initiating ART early and suppressing the virus before significant deterioration of the immune system occurs, preserves immune function to prevent clinical disease progression [5, 6], improves gross motor and neurodevelopmental profiles [7], and can reduce the presence of viral reservoirs in adolescence [8, 9]. Thus, EID is critical to the early identification, treatment, and long term health of these infants. EID also provides reassurance to the mothers of infants who remain HIV negative [10] which has been associated with improved infant bonding [11].

Despite the obvious benefits of receiving EID services, UNAIDS estimates that less than 40 % of HIV-exposed infants actually receive services within their first 2 months of life [12]. Eighteen studies from across East Africa have reported on the proportion and/or age of HIV exposed infants who are enrolled in EID [usually defined as age when a dried blood sample (DBS) was provided] [13–31]. Estimates of uptake from these studies range from 25 % [17] to 88.3 % [15] with age of first DBS ranging from a median of 5.6 weeks [15] to 5 months [17, 21]. While the National AIDS Control Council of Kenya (NACC) estimates that EID coverage in Kenya reached 45 % of eligible infants in 2013 [32], estimates from individual Kenyan studies range widely from a low of 40 % up to a high of 87 % [18, 27, 28]. Median age of enrollment in the few Kenyan studies that tracked it ranged from between 8 weeks to 5 months [21, 27]. The limited coverage and shortcomings of current EID services means that many HIV+ infants are not diagnosed early enough to fully benefit from available treatment [33].

Despite the importance of early detection to signal treatment initiation for HIV+ infants, EID services in East Africa have received considerably less attention than other aspects of PMTCT care. Especially lacking are studies that investigate predictors of “on-time” (infant ≤6 weeks of age) engagement in EID that would help to identify factors that put infants at risk for late engagement and mutable targets for intervention. We could identify only 3 peer-reviewed published studies and one publically available master’s thesis conducted in East Africa [17, 22, 25, 34] that report on predictors of engagement in EID. None were conducted in Kenya. Combined, these studies highlight several potentially important predictors of timely engagement in EID (infant ≤6–8 weeks of age), specifically: mother’s current ART status (being on ART [22, 25]; not being on ART [17]; mother’s engagement in PMTCT during pregnancy [22]); mother’s access to an independent source of income [17]; having ≥2 previous children [25]; having fewer concerns that her child will face discrimination if HIV+ [34]; and delivering in a government facility [22]. While far from definitive, these studies offer some preliminary evidence for the importance of these predictors in timely EID engagement. Nevertheless, more studies are needed. Also lacking are studies that explore predictors of “very late” engagement (≥12 weeks of age) when the benefits of costly ART treatment are compromised. Predictors of really late engagement might be quite different than those that predict early engagement and are critical for the development of outreach and retention efforts.

This study draws on baseline data from an on-going cluster randomized study of an intervention to improve EID services at six sites across Kenya [35]. Potential predictors of “on time” (infant ≤6 weeks of age) EID engagement and “very late” (infant ≥12 weeks of age) are examined in a large and diverse sample.

## Methods

This study sample included 756 HIV-infected mothers and their HIV-exposed infants (mother-infant pairs) enrolled in early infant diagnosis (EID) programs at six Kenyan government hospitals between February 2014 and June 2015. The hospitals ranged in size (provincial, county, and sub-county level), geographic regions (2 western, 2 central and 2 coastal), and population density (3 urban and 3 peri-urban sites). Participants were enrolled in a larger RCT evaluating the impact of the HIV Infant Tracking System (HITSystem) web-based intervention (35; Clinical Trials Id: NCT02072603). The focus of this study, predictors of age at EID enrollment, is neither an outcome of the larger study nor targeted by the HITSystem (a system level documentation and patient tracking system with text reminders for mothers) that seeks to improve communication and accountability of all stakeholders once infants are enrolled in EID services.

### Participant Eligibility and Consent

HIV-infected mothers whose infant is <18 months of age at the time of initial enrollment in EID and presents for care through the maternal and child health (MCH) department at each site were eligible for inclusion in this dataset. Data from mothers <18 years of age or who present for care through other departments (e.g., Pediatric Inpatient Ward) were not included in analyses. Mothers were informed about the study during EID enrollment by trained research or clinical staff, and those wanting to participate provided written informed consent prior to enrollment or completion of the baseline survey. Less than 2 % of mothers declined participation. The study protocol was reviewed and approved by the Institutional Review Boards at the Kenya Medical Research Institute (protocol #2726), and the University of Kansas Medical Center (protocol #13793).

### Procedures

During the first EID enrollment visit, a brief survey was conducted with each consenting mother to collect basic demographic data and other variables of interest including: maternal age, parity, education level, partner status, partner support, income level, disclosure status, HIV stigma, violence, mother’s current HIV care status, how she was informed about EID services, EID knowledge, travel time to hospital and associated costs, and financial concerns. Other data (i.e., infant’s date of birth, antenatal care, maternal ARV prophylaxis regimens, and postpartum infant prophylaxis) was abstracted from patient charts.

### Measures

The dependent and potential predictor variables are described in Table 1.

**Table 1:** Study measures

Measure	Definition/Survey Item	Scaling
*Dependent variable*		
Infant age at DBS collection	Infant age in weeks based on number of days between the date of birth and DBS collection	“On-time”: 0–6 weeks vs. ≥7^a^ “very late”: ≥12 weeks vs. 0–11
*Independent variables*		
Study site^b^	Study site where mother enrolled	
Maternal age	Mother’s current age	Continuous variable; years
Number of children	Number of children currently in mother’s care	Continuous variable; number
Education level	“What is the highest level of education you have completed?”	0 (No formal education), 1 (Partial/completed primary), or 2 (Partial secondary or beyond)^c^
Partner status	“Are you currently with a partner?”	0 (No) or 1 (Yes, regardless of cohabitation status)
Partner/family support^d^	“My partner/family members support my efforts to come to the hospital for my own health” “My partner/family members support my efforts to come to the hospital for my infant’s health”	1 (strongly disagree) through 4 (strongly agree)
Income level	Average weekly income	<750 Kenya Shillings^e^ or ≥750 Kenya Shillings
Disclosure	“To whom have you disclosed your HIV status?”	0 (No one) or 1 (Anyone)
HIV stigma^d^	“Medical staff treat me badly because I am HIV positive” “Medical staff look down on me because I am HIV positive and had a baby”	1 (strongly disagree) through 4 (strongly agree)
Physical/psychological violence	“Has your partner mistreated you physically or psychologically in the past week?” “Has any family member mistreated you physically or psychologically in the past week?”	0 (No to both items) or 1 (Yes to either item)
How mother was informed about EID	“How did you learn about EID?”	0 (anyone else) or 1 (a health care worker while pregnant (PMTCT))
EID knowledge	Five ‘true’ or ‘false’ statements^f^	Correct answers summed to create a 0 (lower) to 5 (higher) knowledge scale
Travel time to hospital	Number of minutes	Converted to hours for modeling
Cost of travel to hospital	Cost of round trip in Kenyan Shillings	Continuous variable; Kenyan Shillings
Concerns about money	“I worry that I won’t have enough money to get to the hospital for my infant’s scheduled HIV tests and re-tests”	1 (strongly disagree) through 4 (strongly agree) Continuous variable

^a^Cut-offs based on findings from the CHER study^3^ that demonstrated the benefits of early ART initiation in reducing mortality and HIV progression

^b^Included as a control variable in the analysis

^c^Based on feedback from our in-country colleagues, the six original educational level categories were collapsed into the more meaningful categories

^d^Responses to items were averaged to create scales used in modeling

^e^Approximately $7.74 USD

^f^Questions related to knowledge about testing and follow-up strategy for HIV testing among infants

### Analyses

The goal of our analyses was to identify predictors of (1) “on-time” (≤6 weeks of age) versus “late” (≥7 weeks) and (2) “on-time” (≤6 weeks) versus “very late” (≥12 weeks) infant testing and quantify their association with the odds of “on-time” or “very late” testing. For descriptive purposes we compared the “on-time” and “late” (tested at or after 7 weeks of age) groups by computing their respective medians and standard deviations on the quantitative variables and frequencies on the categorical variables. Similarly, the “very late” group (a subset of the “late” group) was compared with the rest of the “late” group.

We used logistic regression to examine associations between the variables and the dichotomous dependent variables. Including the full set of variables in a logistic regression model would result in too large a model given the limited numbers in the “late” and “very late” groups and potentially raise multicollinearity concerns. Thus we followed a two-step model selection and fitting process for both dependent variables [36].

First we used the ‘lasso’ (least absolute shrinkage selection operator) [37] to select a subset of candidate predictors for each dependent variable. The lasso is similar to ridge regression in imposing a constraint on the magnitude of the regression coefficients but tends to shrink some coefficients to zero, thereby providing variable selection without the drawbacks of selection methods based on* p* values.

We implemented the lasso for logistic regression using the ‘glmnet’ package in R [38]. All variables except study site (a six-category control variable to be included in the final logistic regression models regardless of association with the dependent variables) were included in the lasso modeling; two dummy variables were created to model education level. For the “on-time” versus “late” model we set the lasso tuning parameter to select 12 candidate predictors, resulting in about 10 late observations per variable (including the site variable, equivalent to five dummy variables) in the final logistic regression model. Given the small size of the “very late” group, we took a less conservative approach and set the tuning parameter in the “very late” model to select seven candidate predictors, resulting in five “very late” observations per variable in the final logistic regression model [39]. Observations (n = 186) with missing data on any variable, including the individual stigma, support, and violence items, were excluded from this step of the analysis, leaving 570 observations in the lasso models for both dependent variables.

Next we fit the final logistic regression model for each dependent variable using the LOGISTIC Procedure in SAS 9.4, including study site and the candidate predictors selected in the lasso step. The lasso yields regression coefficient estimates that are biased toward zero, a limitation that can be addressed by re-fitting the lasso-selected model without the lasso constraint [31]. Observations with missing data on any candidate predictor were excluded from the final models, leaving 630 (with 168 “late”) for the model examining “on time” (≤6 weeks of age) versus “late” and 710 (with 67 “very late”) for the “very late” (≥12 weeks of age) model. Firth regression was used for the “very late” variable to avoid quasi-complete separation.

## Results

Descriptive statistics for the total sample shown in Table 2. Nearly three quarters (73.8 %) of the mothers in our sample were able to secure testing for their infant at or before 6 weeks of age, however over a quarter (25.8 %) were not. Rate of “on time” testing varied greatly by study site. Most sites (4 of 6) had the majority of their infants tested at or before 6 weeks (66–92 %). However, one peri-urban and one urban site evidenced much poorer rates of on-time testing with only 47–59 % of infants getting tested before 7 weeks of age. Average age of mothers who brought their child “on time” versus “late” was very similar, however mothers who came “late” were more likely to: have lower levels of education, not have disclosed their HIV+ status to anyone, not have been on ART before becoming pregnant, not have learned about EID from health care staff during PMTCT, report longer travel time to the hospital, and more concern about having sufficient funds to get to the hospital. The majority of mothers “agreed” or “strongly agreed” that their partner/family members support their efforts to come to the hospital for their infant’s health (“on time” 85 %, “late” 73 %), but more “late” mothers “disagreed” or “strongly disagreed” (32 %) with this statement than mothers who were “on time” (21 % *p* > 0.05; not displayed in Table 2). Similarly, the majority of mothers “disagreed” or “strongly disagreed” with the statement that “Medical staff look down on me because I am HIV positive and had a baby” (“on time” 87 %, “late” 86 %), but more “late” mothers “agreed” or “strongly agreed” (5 %) than mothers who were “on time” (2 % *p* < 0.04; not displayed in Table 2).

**Table 2:** Characteristics of maternal/infant pairs who completed “on time” or “late” infant testing February 2013 to June 2014

Variable	Total sample (n = 756)	“On-time” <6 weeks of age (n = 552)	“Late” ≥7 weeks of age (n = 204)	*p* value	“Very late” ≥12 weeks age (n = 73)
		N (%)	N (%)		
Study site				<0.0001	
Peri-urban site	134	100 (74.6)	34 (25.4)		15
Urban site	115	66 (57.4)	49 (42.6)		18
Peri-urban site	62	34 (54.8)	28 (45.2)		17
Urban site	276	225 (81.5)	51 (18.5)		15
Peri-urban site	58	50 (86.2)	8 (13.8)		0
Urban site	111	77 (69.4)	34 (30.6)		8
Maternal age [median (IQR)]	756	29 (25–34)	29 (24–33)	0.139^a^	27 (23–32)
Education level				0.0004	
No formal education	46	26 (56.5)	20 (43.5)		8
Partial/completed primary	389	271 (69.7)	118 (30.3)		44
Partial secondary/beyond partial/University	306	244 (79.7)	62 (20.3)		18
Partner status				0.092	
Yes and live together	525	394 (75)	131 (25)		41
Yes but don’t live together/no current partner	206	142 (68.9)	64 (31.1)		28
Income level				0.434	
<750 KSH	406	292 (71.9)	114 (28.1)		46
≥750 KSH	295	220 (74.6)	75 (25.4)		22
Disclosure				0.014	
Disclosed to anyone	666	497 (74.6)	169 (25.4)		55
Has not disclosed to anyone	57	34 (59.7)	23 (40.4)		13
Violence				0.112	
Partner/family member mistreated in past week	143	112 (78.3)	31 (21.7)		10
No	595	427 (71.8)	168 (28.2)		58
Mother’s ART status prior to pregnancy				0.005	
Already on HAART before pregnancy	297	232 (78.1)	65 (21.9)		15
No	345	236 (68.4)	109 (31.6)		43
How mother was informed about EID				<0.0001	
By health care worker during PMTCT/pregnant	628	477 (76)	151 (24)		46
All others	116	66 (56.9)	50 (43.1)		24
EID knowledge [median (IQR)]	745	4 (3–5)	4 (3–5)	0.618^a^	4 (3–5)
Travel time to hospital [minutes, median (IQR)]	721	30 (30–60)	60 (30–60)	0.046^a^	60 (30–90)
Travel costs to hospital [KSH, median (IQR)]	733	120 (100–200)	100 (100–200)	0.910^a^	100 (100–200)
Concerns about money				0.021	
Strongly disagree	284	224 (78.9)	60 (21.1)		15
Disagree	173	124 (71.7)	49 (28.3)		17
Agree	123	86 (69.9)	37 (30.1)		13
Strongly agree	162	107 (66)	55 (34)		25

*Note* Denominator changes due to missing values. Results of analyses contrasting the “very late” subset with the rest of the mothers in the “late” group (n = 131) are presented in the text

*KSH* Kenyan Shillings

^a^Two-sided *p* value for Wilcoxon two-sample test

When compared to the rest of the mothers in the “late” group (n = 131), mothers in the “very late” (n = 73) subset were less likely to have sought services in an urban site (56 vs. 71 %, *p* = 0.033), disclosed to anyone (81 vs. 92 %, *p* = 0.024), been on ART prior to pregnancy (26 vs. 43 %, *p* = 0.027), and learned about EID from a health care worker (66 vs. 80 %, *p* = 0.024). The “very late” group also had a longer median travel time [60 min (IQR 30–90) vs. 30 min (30–60), *p* = 0.009] and higher median scores on the money worries item [3 (IQR 2–4) vs. 2 (1–3), *p* = 0.026]. The two groups were similar on the remaining descriptive variables in Table 2.

### Logistic Regressions

Of the variables analyzed in the lasso stage, the following 12 were selected as candidate predictors for the model examining “on time” versus “late” infant testing: education level (two dummy variables), maternal age, travel time to hospital, concern about money for transportation, disclosure, how mother was informed about EID, stigma, social support, violence, income, and partner status. The seven candidate predictors selected in the lasso step for “very late” enrollment were education level (two dummy variables), maternal age, travel time to hospital, concern about money for transportation, disclosure, how mother was informed about EID, and social support.

Results of the final logistic regression models are provided in Tables 3 and 4. Area under the ROC curve was 0.72 for the “on time” model and 0.79 for the “very late” model, indicating moderate to good predictive power.

**Table 3:** Regression results from model for “on-time” versus all late enrollment

Variable	Chi square	df	*p* value	aOR	95 % CI for aOR
Site	25.99	5	<0.001		
Education	9.98	2	0.007		
At least partial primary^a^	4.06	1	0.044	2.19	1.02–4.71
At least partial secondary^a^	8.91	1	0.003	3.54	1.54–8.11
Mother’s age	1.26	1	0.262	1.02	0.99–1.06
Travel time (hours)	2.07	1	0.150	0.80	0.60–1.08
Money worries^b^	3.64	1	0.056	0.81	0.65–1.01
Any disclosure	1.66	1	0.198	1.65	0.78–3.55
How informed about EID	18.35	1	<0.001	3.05	1.83–5.09
Stigma^b^	5.46	1	0.019	0.78	0.64–0.96
Social support^b^	1.15	1	0.283	1.13	0.91–1.40
Violence (any)	3.47	1	0.063	1.71	0.97–3.01
Income (750+)	0.41	1	0.525	0.87	0.56–1.34
Has partner	0.18	1	0.668	0.87	0.47–1.63

*Note* All late = “late” and “very late”

^a^Referent is no formal education

^b^Standardized

**Table 4:** Regression results from model for “very late” vs. all other enrollment

Variable	Chi square	df	*p* value	aOR	95 % CI for aOR
Site	12.37	5	0.030		
Education	4.70	2	0.095		
Mother’s age	2.86	1	0.091	0.96	0.92–1.01
Travel time (hours)	4.84	1	0.028	1.50	1.05–2.14
Money worries^a^	1.65	1	0.199	1.22	0.90–1.64
How informed about EID	15.09	1	<0.001	0.31	0.17–0.56
Social support^a^	0.72	1	0.396	0.89	0.67–1.17

*Note* All other = “on-time” and “late” enrollment

^a^Standardized

### Predictors of “On-time” Infant Testing

At α = 0.05 there were five statistically significant predictors of “on-time” testing (in addition to study site, included as a control variable). The strongest predictor was how the mother was informed about EID, with those informed by a health care worker during pregnancy having three times the odds of “on-time” testing as those informed by another source. The odds of on-time testing were about twice as high for mothers with at least partial primary education (vs. no formal education), and 3.5 times as high for those with at least partial secondary education. Higher scores on the stigma measure were associated with lower odds of “on-time” testing, with a reduction in odds of 22 % per 1-SD higher score on the stigma scale.

### Predictors of “Very Late” Infant Testing

As with “on-time” vs. “late” testing, the strongest predictor of “very late” (≥12 weeks of age) testing was how the mother was informed about EID; odds of “very late” testing were nearly 70 % lower for mothers informed by a health care worker during pregnancy. Odds of “very late” testing were 1.5 times higher per additional hour of reported travel time to the hospital. Study site was the only other statistically significant predictor of “very late” testing.

## Discussion

This study explored rates and predictors of “on time” versus “late” and “very late” engagement in EID services among a large sample of mother-infants dyads seeking care at six health care facilities in Kenya. Consistent with other East African studies [14, 16, 24], nearly three quarters of mothers secured EID testing for their infant before 7 weeks of age. Nevertheless, more than a quarter of infants who needed testing did not receive it in a timely manner. Delays in initiation of EID testing compromise the effectiveness of life sustaining ART treatment for HIV-infected infants [4, 40] and contribute to poor health outcomes in this vulnerable population [8]. Early infant testing is also a cornerstone in achieving the global goals of closing the treatment gap for eligible infants and preventing child mortality due to HIV [41, 42].

Descriptive analyses revealed significant variation of “on time” testing by clinic site with 2 of 6 facilities reporting that only about half of their infants had been tested before 7 weeks. Why these specific clinics (one peri-urban, one urban from different regions) struggled to achieve testing rates seen in other similar facilities is unknown, but we assume they are specific to the sites themselves and as such we controlled for site in the logistic regression analyses. While not directly examined in this study, we have observed this same pattern in our experience working to scale-up novel system-level interventions across multiple sites. Facilities often have their own unique barriers (e.g., lack of space, consistent medication supply and/or poor coordination between different clinics), cultures (e.g., stigmatizing beliefs, established procedures that contradict care guidelines) and staffing challenges (e.g., providers who lack knowledge or motivation) that decrease the likelihood of meeting clinical guideline goals [43]. Future studies should explore site-specific barriers to EID engagement to better inform needed changes.

Results of the logistic regression analyses revealed that mothers who were informed about EID services by a healthcare provider during their pregnancies had three times the odds of bringing their infant for testing before 7 weeks of age as mothers who learned about EID from other sources. Clearly mothers had to be engaged in PMTCT and/or other prenatal care to have had the opportunity to be informed about EID during their pregnancies which has been highlighted in prior research [22]. This finding underscores the critical role that healthcare providers play in engaging women early and retaining them throughout their pregnancies to ensure that they are linked to EID services after giving birth. Ensuring continuity of care is critical, yet in most settings prenatal care, delivery, EID and HIV care services are provided by separate provider groups who are located in several different clinics often housed in different sections of health centers. This puts the burden on mothers to stay engaged in care by finding their way to the next clinic, establishing new relationships with new providers, and adapting to a new setting and schedule. Our findings reinforce other work [16, 23, 44, 45] that has highlighted the need for co-locating services and linking mothers to the next clinic or provider group as essential to improving “on-time” EID service utilization. Other options include task shifting [46, 47] so that the same staff follow mothers all the way from PMTCT to EID services and/or outreach [48, 49] to mothers during transitions to ensure that they stay engaged.

Mothers with less formal education were found to be at increased risk for seeking “late” EID testing. Like findings in PMTCT engagement and retention [50–52], mothers with less education experienced a whole host of additional barriers to seeking care, which are also likely at play here. Additional outreach and retention efforts focused on engaging these mothers will be necessary.

Mothers who perceived less stigma from health care providers for being HIV+ and having a baby were considerably more likely to seek “on-time” testing for their infant. This finding is consistent with other studies that have demonstrated the negative impact of perceived stigma on EID treatment engagement [34], sharing of fertility desires [53, 54], and ART adherence [55]. It also highlights the importance of ensuring that all providers send a consistent and supportive message about childbearing options to prevent patient disengagement from care which in turn leads to worse outcomes for mother, partners and infants [56, 57].

Predictors of “very late” (≥12 weeks of age) infant testing included being informed by a healthcare provider about EID during pregnancy and travel time to the hospital. Like the earlier “on-time” findings, results of this analysis highlight the critical role that providers play with the odds of seeking “very late” infant testing being 70 % lower for mothers who were informed by a healthcare provider while they were pregnant. Not surprisingly, living farther away from the hospital greatly contributed to “very late” seeking of EID services. Enhancing options for mothers who live great distances from EID services, like increasing the number of EID clinics and/or developing mobile EID services, would address this known barrier. However, limited resources in an already overburdened Kenyan healthcare system limit the likelihood of these options coming to fruition. Other more cost effective approaches, like small stipends for mothers to pay for transport and accommodations at or near the hospitals might be effective and are already being used to support other health behaviors (e.g., ART adherence) [58]. At the very least, providers should start early to develop a plan with women to facilitate *early* infant testing.

This study makes an important and novel contribution to the literature, but it is not without its limitations. Chief among them are that all of the mothers in this study did eventually present for EID services. We do not have any information about mothers who never sought services, nor do we know if the group of users of the services in this study represents a small or large proportion of the total number of mothers of HIV-exposed infants. Future studies should explore what proportion of all HIV-exposed infants receive EID services, as well as, what predicts non-engagement.

## Conclusion

This study found that a quarter of infants in EID services at six Kenyan hospitals failed to get tested “on time” (before 7 weeks of age). Findings highlight the importance of ensuring that health care providers actively and repeatedly inform HIV+ mothers of the availability of EID services, reduce stigma by frequently communicating judgment free support, and assist mothers in early travel planning to access EID services after their babies are born. Extra care should be focused on engaging mothers with less formal education who are at increased risk for seeking “late” EID testing. Getting all HIV exposed infants engaged in EID services and tested before 6 weeks is critical to optimize HIV pediatric outcomes. This study offers clear targets for improving outreach and service integration that could help to make this important goal a reality.
